# The Kynurenine Pathway Metabolites in Cord Blood Positively Correlate With Early Childhood Adiposity

**DOI:** 10.1210/clinem/dgac078

**Published:** 2022-02-12

**Authors:** Karen Mei-Ling Tan, Mya-Thway Tint, Narasimhan Kothandaraman, Navin Michael, Suresh Anand Sadananthan, S Sendhil Velan, Marielle V Fortier, Fabian Yap, Kok Hian Tan, Peter D Gluckman, Yap-Seng Chong, Mary F F Chong, Yung Seng Lee, Keith M Godfrey, Johan G Eriksson, David Cameron-Smith

**Affiliations:** 1 Singapore Institute for Clinical Sciences (SICS), Agency for Science, Technology and Research (A*STAR), 117609, Singapore; 2 Department of Laboratory Medicine, National University Hospital, 119074, Singapore; 3 Department of Obstetrics and Gynaecology, Human Potential Translational Research Programme, Yong Loo Lin School of Medicine (YLLSOM), National University of Singapore, 119228, Singapore; 4 Institute of Bioengineering and Bioimaging (IBB), Agency for Science Technology and Research, 138669, Singapore; 5 Department of Diagnostic and Interventional Imaging, KK Women’s and Children’s Hospital, 229899, Singapore; 6 Duke-National University of Singapore (NUS) Medical School, 169857, Singapore; 7 Department of Pediatric Endocrinology, KK Women’s and Children’s Hospital, 229899, Singapore; 8 Lee Kong Chian School of Medicine, Nanyang Technological University, 636921, Singapore; 9 Perinatal Audit and Epidemiology, Department of Maternal Fetal Medicine, KK Women’s and Children’s Hospital, 119228, Singapore; 10 Liggins Institute, University of Auckland, Auckland 1023, New Zealand; 11 Yong Loo Lin School of Medicine (YLLSOM), National University of Singapore, 117597, Singapore; 12 Saw Swee Hock School of Public Health, National University of Singapore and National University Health System, 117549, Singapore; 13 Department of Paediatrics, Yong Loo Lin School of Medicine, National University of Singapore, 119228, Singapore; 14 Khoo Teck Puat – National University Children’s Medical Institute, National University Health System, 119074, Singapore; 15 MRC Lifecourse Epidemiology Unit, University of Southampton, Southampton SO16 6YD, United Kingdom; 16 NIHR Southampton Biomedical Research Centre, University of Southampton Hospital, Southampton SO16 6YD, United Kingdom; 17 Folkhälsan Research Center, 00250 Helsinki, Finland; 18 Department of General Practice and Primary Health Care, University of Helsinki, 00290 Helsinki, Finland; 19 Department of Biochemistry, Yong Loo Lin School of Medicine, National University of Singapore, 117596, Singapore

## Abstract

**Context:**

The kynurenine pathway generates metabolites integral to energy metabolism, neurotransmission, and immune function. Circulating kynurenine metabolites positively correlate with adiposity in children and adults, yet it is not known whether this relationship is present already at birth.

**Objective:**

In this prospective longitudinal study, we investigate the relationship between cord blood kynurenine metabolites and measures of adiposity from birth to 4.5 years.

**Methods:**

Liquid chromatography–tandem mass spectrometry was used to quantify cord blood kynurenine metabolites in 812 neonates from the Growing Up in Singapore Towards healthy Outcomes (GUSTO) study. Fat percentage was measured by air displacement plethysmography and abdominal adipose tissue compartment volumes; superficial (sSAT) and deep subcutaneous (dSAT) and internal adipose tissue were quantified by magnetic resonance imaging at early infancy in a smaller subset of neonates, and again at 4 to 4.5 years of age.

**Results:**

Cord blood kynurenine metabolites appeared to be higher in female newborns, higher in Indian newborns compared with Chinese newborns, and higher in infants born by cesarean section compared with vaginal delivery. Kynurenine, xanthurenic acid, and quinolinic acid were positively associated with birthweight, but not with subsequent weight during infancy and childhood. Quinolinic acid was positively associated with sSAT at birth. Kynurenic acid and quinolinic acid were positively associated with fat percentage at 4 years.

**Conclusion:**

Several cord blood kynurenine metabolite concentrations were positively associated with birthweight, with higher kynurenic acid and quinolinic acid correlating to higher percentage body fat in childhood, suggesting these cord blood metabolites as biomarkers of early childhood adiposity.

Tryptophan (TRP) is the precursor of multiple metabolites and hormones including serotonin, melatonin, and kynurenine (KYN) (1, 2). KYN is the first component of a major TRP metabolic pathway involved in regulation of immunity, metabolism, and excitatory neurotransmission (1-4). In the KYN pathway (5), TRP is first converted to KYN by the rate-limiting enzymes indoleamine-2,3-dioxygenase (IDO) and tryptophan-2,3-dioxygenase (TDO) (1-3, 5). Under normal physiological conditions, the majority of KYN is produced by TDO in the liver, with glucocorticoids being key regulators of this pathway (3). However, in obesity the KYN/TRP ratio is increased, with inflammatory cytokine activation of IDO (6, 7). IDO is expressed in many tissues, including the placenta, and increased KYN/TRP ratio, indicative of increased IDO activity, has been reported in obese pregnant women (1-3, 8).

KYN can undergo further metabolism via 3 possible enzymatically regulated pathways, generating either kynurenic acid (KA), 3-hydroxykynurenine (HK), or anthranilic acid (AA). HK can undergo further metabolism to form xanthurenic acid (XA) or alternatively can be converted to 3-hydroxyanthranilic acid (HAA) and quinolinic acid (QA). QA is a precursor required in the synthesis of nicotinamide adenine dinucleotide (NAD^+^). For both XA and QA, the first step in synthesis is catalyzed by kynurenine-3-monooxygenase (KMO) to generate HK, hence HK/KYN ratio is a proxy for KMO activity (9). Increased flux through KMO is reported in obesity (7), with increased circulatory concentrations of XA and QA reported in individuals with insulin resistance and increased cardiovascular disease risk (10-15). However, there are limited data regarding the biological roles exerted by XA and QA in obesity. XA has zinc chelation properties, with a possible capacity to inhibit insulin secretion (16), while QA is an N-methyl-D-aspartate (NMDA) receptor agonist and has been identified as a risk factor in several psychiatric disorders such as depression and schizophrenia that are frequently associated with obesity (2, 17, 18).

TRP requirements are increased during pregnancy (5), with KYN metabolites at several fold higher concentrations in umbilical cord plasma than in maternal plasma (19). The roles and functions of cord blood KYN (KYN_CB_) metabolites are complex, with evidence for critical function in many aspects for fetal survival and growth, including vasodilatory function in the cord and placenta vessels, antioxidative capacity, regulation of poly-ADP ribose polymerase activity, fetal energetics via NAD + synthesis, and T cell differentiation (5, 20). However, given the complexity and importance of KYN_CB_ metabolite function for fetal growth and survival, and the established association with obesity in later life, few studies have addressed the relationship of KYN_CB_ with early life adiposity measures. We hypothesized that concentrations of metabolites in the KYN pathway would be associated with adiposity at birth and in early life. This study reports TRP and KYN metabolite concentrations in antenatal blood and umbilical cord blood in mothers and neonates from the Growing Up in Singapore Towards healthy Outcomes (GUSTO) cohort, a prospective Asian mother-offspring cohort study (21). The association of these metabolites with maternal health, including body mass index (BMI) and gestational blood glucose, are also examined to explore possible transgenerational influences. To the best of our knowledge, this is the first study to systematically examine the association between KYN_CB_ metabolites and early life adiposity to determine whether KYN_CB_ metabolites could be early biomarkers of adiposity in early childhood.

## Methods

### Study Population

GUSTO is a prospective mother-offspring cohort study (21). Pregnant women aged 18 years and older, from the 3 major ethnic groups in Singapore—Chinese, Malay, and Indian—were recruited during the first trimester of pregnancy from 2 public maternity units in Singapore—KK Women’s and Children’s Hospital and National University Hospital—from 2009 to 2010. All participants gave written informed consent for their and their offspring’s participation in this study. The study was conducted according to the guidelines laid down in the Declaration of Helsinki. Ethical approval was obtained from the Domain Specific Review Board of Singapore National Healthcare Group (reference D/09/021) and the Centralised Institutional Review Board of SingHealth (reference 2009/280/D). A flow chart of the study is shown in Supplementary Figure 1 (22).

### Antenatal and Cord Blood TRP-KYN Metabolite Measurements

Fasting antenatal blood was collected from pregnant women at 26-28 weeks gestation from a peripheral vein into EDTA tubes. Umbilical cord blood was collected at delivery using a syringe from the umbilical vein into EDTA tubes. Within 2 hours, blood was centrifuged at 1600*g* for 10 minutes at 4 °C to obtain plasma. Plasma was further centrifuged at 16 000*g* for 10 minutes at 4 °C and stored at −80 °C for later analyses. TRP and KYN metabolites were quantified by liquid chromatography–tandem mass spectrometry (LC-MS/MS) at BEVITAL AS, Norway, as described previously (23). The coefficients of variation (CV) of a plasma control run in duplicates over 10 plates ranged between 3.3% to 8.4% for the KYN metabolites. Cord blood plasma metabolites were all measured in one batch and antenatal plasma metabolites were all measured in one batch.

### Maternal Characteristics and Measurements

Self-reported age, ethnicity, and prepregnancy weights of mothers were recorded. Prepregnancy BMI was calculated from the self-reported prepregnancy weight and measured height at booking. Pregnant women underwent a 2-hour 75g oral glucose tolerance test (OGTT) at 26-28 weeks gestation. Glucose concentrations were measured using a hexokinase method (Advia 2400 Chemistry system, Siemens Medical Solutions Diagnostics) and Beckman LX20 Pro analyzer (Beckman Coulter).

### Neonate and Child Characteristics and Measurements

Gestational age was determined based on fetal measurements from the first trimester ultrasound scans. The estimated fetal weight (EFW) at 26-28 weeks gestation was obtained from fetal biometry assessments using the Hadlock-4 formula (24), as follows: Log10 weight = 1.3596 − 0.00386 abdominal circumference (AC) × femur length (FL) + 0.0064 head circumference (HC) + 0.00061 biparietal diameter × AC + 0.0424 AC + 0.174 FL. The EFWs were assigned a bulk centile using the GROW software2 (www.gestation.net) (25). The centile calculation included adjustments for the following nonpathological factors: maternal height, weight, and parity; fetal sex; and exact gestational age on day of ultrasound scan. No customization was performed for ethnicity. An EFW centile < 10th centile was used to identify intrauterine growth restriction (IUGR) (26). Data on mode of delivery and birth weight were transcribed from hospital medical records. Body composition was measured in a subset of the children by air displacement plethysmography using the PEA POD Infant Body Composition System Version 3.1.0 (Cosmed, Italy) at birth (n = 255) (27, 28), and BOD POD body composition tracking system version 5.2.0 (Cosmed, Italy) at 4 years (n = 224) (29).

Abdominal magnetic resonance imaging (MRI) was performed in a subset of neonates within 2 weeks after delivery (n = 262) (30), and at age of 4.5 years (n = 214) (31). Abdominal adipose tissue compartment volumes were used as a measure of abdominal adiposity. The segmentation and quantification of abdominal adipose tissue compartment volumes in the neonatal period and at 4.5 years were described previously in detail (31, 32). The abdominal adipose tissue compartment volumes were categorized into superficial subcutaneous adipose tissue (sSAT), deep subcutaneous adipose tissue (dSAT) and internal adipose tissue (IAT) (30) at the neonatal period. Visceral adipose tissue (VAT), that is, fat around the abdominal organs such as liver, mesenteric, or omental, was minimal in the neonatal period, thus described as IAT. However, VAT was substantially greater at 4.5 years, thus IAT was described as VAT accordingly (31).

### Statistical Analyses

Antenatal KYN (KYN_AN_) and cord blood KYN (KYN_CB_) metabolite concentrations were transformed into standardized scores (Z-scores) to compare the strengths of associations across metabolites. The correlations between maternal antenatal and cord blood KYN metabolites were studied using Pearson correlation. Multiple regression analyses were performed to determine the associations between maternal factors (age, ethnicity, prepregnancy BMI, and maternal plasma glucose) and KYN_AN_ metabolites concentrations. ANOVA was used to determine the statistical significance of the difference in study characteristics between those without and with fat percentage and abdominal adipose tissue volume measurements for continuous variables and Chi-square test for categorical variables.

Multiple variable regression analyses were performed to determine the associations between maternal and perinatal factors (ethnicity, maternal age, prepregnancy BMI, maternal plasma glucose, delivery mode, gestational age, IUGR, child’s sex, study site) and KYN_CB_ metabolites concentrations. Neonate and child adiposity measures such as weights, body fat percentage from PEA POD and BOD POD, and sSAT, dSAT, and IAT/VAT were also converted to Z-scores as outcomes. Multivariable regression analyses were performed to examine the associations between KYN_CB_ metabolites and child’s adiposity measures adjusting for covariates; sex, ethnicity, study site, duration of gestation, IUGR, delivery mode, prepregnancy BMI and maternal plasma glucose. *P* values were corrected using the Benjamini-Hochberg method with false discovery rate (FDR) of 0.05(25). Statistical analyses were performed with SPSS Statistics for Windows, Version 25.0. (IBM Corp., Armonk, NY).

## Results

The characteristics of the study cohort, included in this analysis, are shown in Table 1. The participants were children of Chinese, 399 (49.1%), Malay 247 (30.4%), and Indian 166 (20.4%) ethnicities. There were 432 (53.2%) male neonates and 380 (46.8%) female neonates. Girls were lighter at birth than boys but had greater body fat and subcutaneous adipose tissue volumes as neonates (girls 10.7% vs boys 9.5%) and at age 4.5 years (girls 26.0% vs boys 24.8%). Boys had higher VAT at age 4.5 years (Table 1). The comparison between subjects with and without fat percentage and abdominal adipose tissue volume measurements is shown in Supplementary Table 1 (22). Neonates with fat percentage and abdominal adipose tissue volume measurements had younger mothers with lower antenatal 2-hour post-OGTT plasma glucose concentrations. There were more Malay neonates with fat percentage and abdominal adipose tissue volume measurements performed, and more neonates born by vaginal delivery with fat percentage measurements. Birthweight and gestational age were similar between those with and without fat percentage and abdominal tissue volume measurements, as were the cord blood kynurenine metabolite concentrations.

**Table 1.** Characteristics of the study cohort

Characteristics	N	All	N	Boys	N	Girls
Ethnicity	812		432		380	
Chinese	399	49.1%	206	47.7%	193	50.8%
Malay	247	30.4%	138	31.9%	109	28.7%
Indian	166	20.4%	88	20.4%	78	20.5%
Maternal age	812	30.2 (5.2)	432	30.4 (5.1)	380	29.9 (5.4)
Prepregnancy BMI (kg/m^2^)	733	22.9 (4.6)	386	22.7 (4.3)	347	23.2 (4.9)
Antenatal fasting plasma glucose (mmol/L)	771	4.4 (0.5)	409	4.3 (0.5)	362	4.4 (0.5)
Antenatal 2h post-OGTT plasma glucose (mmol/L)	771	6.5 (1.5)	409	6.5 (1.6)	362	6.5 (1.4)
Mode of delivery	812		432		380	
Cesarean section	252	31.0%	135	31.3%	117	30.8%
Vaginal delivery	560	69.0%	297	68.8%	263	69.2%
Duration of gestation (weeks)	812	38.8 (1.4)	432	38.7 (1.3)	380	38.8 (1.4)
Birthweight (kg)	812	3.1 (0.4)	432	3.1 (0.4)	380	3.1 (0.4)
Neonatal fat percentage by PEA POD (%)	255	10.0 (3.5)	132	9.5 (3.4)	123	10.7 (3.6)
Neonatal abdominal adipose tissue volume	262		143		119	
sSAT (mL)	262	77.9 (22.1)	143	73.4 (20.0)	119	83.3 (23.5)
dSAT (mL)	262	13.5 (5.9)	143	12.5 (5.5)	119	14.7 (6.2)
IAT (mL)	262	23.0 (7.9)	143	22.7 (8.1)	119	23.4 (7.8)
Weight at 3 months (kg)	711	6.1 (0.8)	380	6.4 (0.8)	331	5.8 (0.7)
Weight at 6 months (kg)	676	7.7 (1.0)	359	8.0 (1.0)	317	7.4 (0.8)
Weight at 9 months (kg)	648	8.6 (1.0)	347	8.9 (1.0)	301	8.3 (0.9)
Weight at 1 year (kg)	661	9.4 (1.1)	351	9.6 (1.1)	310	9.1 (1.0)
Weight at 1.5 years (kg)	632	10.8 (1.4)	340	11.0 (1.4)	292	10.5 (1.3)
Weight at 2 years (kg)	641	12.0 (1.6)	345	12.2 (1.6)	296	11.7 (1.6)
Weight at 3 years (kg)	644	14.3 (2.2)	351	14.5 (2.2)	293	14.0 (2.0)
Weight at 4 years (kg)	600	16.5 (2.8)	319	16.7 (2.8)	281	16.3 (2.8)
Weight at 4.5 years (kg)	613	17.5 (3.1)	323	17.7 (3.2)	290	17.3 (3.0)
Body fat percentage by BOD POD at 4 years	224	25.4 (7.0)	111	24.8 (6.4)	113	26.0 (7.6)
Abdominal adipose volume by MRI at 4.5 years	214		101		113	
sSAT (mL)	214	414.4 (235.7)	101	381.0 (234.0)	113	444.3 (228.7)
dSAT (mL)	214	162.6 (171.7)	101	141.2 (166.8)	113	181.7 (174.5)
VAT (mL)	214	190.1 (75.1)	101	199.4 (80.3)	113	181.9 (61.5)

Data shown are N (%) for categorical variables and mean (SD) for continuous variables.

Abbreviations: dSAT, deep subcutaneous adipose tissue volume; IAT, internal adipose tissue volume; MRI, magnetic resonance imaging; OGTT, oral glucose tolerance test; sSAT, superficial subcutaneous adipose tissue volume; VAT, visceral adipose tissue volume.

### Determinants of Umbilical Cord Blood KYN Metabolite Concentrations

All KYN metabolites in maternal blood positively correlated with cord blood concentrations (R = 0.19-0.47; all *P* values < 0.001) (Table 2), with cord blood TRP and KYN metabolite concentrations from 1.8- to 19-fold higher than maternal mid-gestation concentrations (Table 2). Chinese women had higher mean (SD) antenatal TRP (TRP_AN_) concentrations (47.8 [7.5] µmol/L) than Malay (44.7 [8.5] µmol/L) and Indian women (44.7 [7.8] µmol/L) (Supplementary Table 2a, Supplementary Table 3 (22)). Indian and Malay women had higher antenatal KYN_AN_ and HK_AN_ concentrations as well as a greater KYN_AN_/TRP_AN_ ratio, compared to Chinese women (Supplementary Table 2a, Supplementary Table 3 (22)). Older maternal age was associated with lower TRP_AN_ concentrations in the pregnant women (Supplementary Table 3 (22)).

**Table 2.** Concentrations of antenatal and cord blood TRP-KYN metabolites and ratios

Metabolite	Antenatal 26-28 weeks (n = 695)	Cord blood (n = 812)	Pearson correlation	*P* value	Corrected *P* value
Tryptophan (TRP) (µmol/L)	46.1 (8.0)	74.1 (12.2)	R = 0.19	<0.001	<0.001
Kynurenine (KYN) (µmol/L)	1.0 (0.2)	3.4 (0.6)	R = 0.24	<0.001	<0.001
Kynurenic acid (KA) (nmol/L)	18.0 (6.6)	350.0 (108.3)	R = 0.26	<0.001	<0.001
3-hydroxykynurenine (HK) (µmol/L)	49.8 (19.3)	111.6 (46.7)	R = 0.29	<0.001	<0.001
Xanthurenic acid (XA) (nmol/L)	11.0 (6.2)	34.0 (13.7)	R = 0.20	<0.001	<0.001
Hydroxyanthranilic acid (HAA) (nmol/L)	72.1 (19.0)	762.4 (269.0)	R = 0.20	<0.001	<0.001
Quinolinic acid (QA) (nmol/L)	381.2 (100.8)	1203.1 (297.7)	R = 0.47	<0.001	<0.001
KYN/TRP ratio *100	2.3 (0.6)	4.6 (1.0)	R = 0.29	<0.001	<0.001
HK/KYN ratio	48.1 (16.1)	33.3 (13.3)	R = 0.23	<0.001	<0.001

Data are represented as mean (SD). R represents the Pearson correlation coefficient. *P* values were determined using Pearson correlation. Corrected *P* values were obtained using Benjamini-Hochberg correction for multiple testing.

Maternal prepregnancy BMI was positively associated with increased antenatal KA (KA_AN_) and XA_AN_ concentrations, but inversely associated with increased antenatal HAA_AN_ concentrations (Supplementary Table 3) (22). Higher maternal prepregnancy BMI was associated with higher cord blood TRP_CB_, KA_CB_, and XA_CB_ concentrations (Table 3). Higher maternal antenatal fasting plasma glucose concentrations were associated with higher antenatal HAA_AN_ and QA_AN_ concentrations and a higher antenatal KYN_AN_/TRP_AN_ ratio (Supplementary Table 3) (22), and the same trend was observed for cord blood QA_CB_ and KYN_CB_/TRP_CB_ (Table 3). Higher maternal antenatal 2-hour post-OGTT plasma glucose concentrations were associated with higher antenatal XA_AN_ and HAA_AN_ concentrations (Supplementary Table 3) (22). However, higher maternal antenatal 2-hour post-OGTT plasma glucose concentrations were associated with lower cord blood KA_CB_ and HAA_CB_ concentrations (Table 3).

**Table 3.** The associations of maternal and perinatal factors with cord blood TRP-KYN metabolite concentrations and ratios

Variable	TRP_CB_	KYN_CB_	KA_CB_	HK_CB_	XA_CB_	HAA_CB_	QA_CB_	KYN_CB_/TRP_CB_	HK_CB_/KYN_CB_
Indian (n = 129) vs Chinese (n = 330)	−0.17 (−0.38, 0.04) *P* = 0.104	0.54 (0.35, 0.74) ***P* < 0.001**	0.51 (0.30, 0.71) ***P* < 0.001**	0.32 (0.11, 0.53) ***P* = 0.003**	0.40 (0.20, 0.60) ***P* < 0.001**	−0.09 (−0.29, 0.11) *P* = 0.376	0.23 (0.02, 0.43) ***P* = 0.031**	0.59 (0.39, 0.79) ***P* < 0.001**	0.09 (−0.13, 0.30) *P* = 0.427
Corrected *P* value	*P* = 0.174	***P* < 0.001**	***P* < 0.001**	***P* = 0.013**	***P* < 0.001**	*P* = 0.417	*P* = 0.077	***P* < 0.001**	*P* = 0.854
Malay (n = 194) vs Chinese (n = 330)	−0.16 (−0.35, 0.03) *P* = 0.099	0.08 (−0.10, 0.26) *P* = 0.377	0.07 (−0.11, 0.26) *P* = 0.433	0.22 (0.03, 0.40) ***P* = 0.022**	−0.12 (−0.30, 0.07) *P* = 0.206	0.25 (0.07, 0.43) ***P* = 0.006**	0.19 (0.00, 0.37) ***P* = 0.045**	0.15 (−0.03, 0.33) *P* = 0.107	0.24 (0.04, 0.43) ***P* = 0.016**
Corrected *P* value	*P* = 0.174	*P* = 0.538	*P* = 0.542	*P* = 0.056	*P* = 0.284	***P* = 0.018**	*P* = 0.091	*P* = 0.178	*P* = 0.079
Maternal age (n = 653)	0.02 (0.01, 0.04) ***P* = 0.006**	0.02 (0.01, 0.03) ***P* = 0.005**	0.00 (−0.01, 0.02) *P* = 0.960	−0.01 (−0.02, 0.07) *P* = 0.386	0.01 (−0.00, 0.03) *P* = 0.131	0.01 (−0.01, 0.02) *P* = 0.204	0.01 (−0.00, 0.03) *P* = 0.122	0.00 (−0.01, 0.02) *P* = 0.811	−0.02 (−0.03, 0.00) *P* = 0.051
Corrected *P* value	***P* = 0.020**	***P* = 0.018**	*P* = 0.960	*P* = 0.552	*P* = 0.219	*P* = 0.291	*P* = 0.136	*P* = 0.811	*P* = 0.171
Prepregnancy BMI (n = 653)	0.03 (0.01, 0.05) ***P* = 0.002**	0.02 (−0.00, 0.03) *P* = 0.087	0.03 (0.02, 0.05) ***P* < 0.001**	0.01 (−0.01, 0.03) *P* = 0.323	0.03 (0.02, 0.05) ***P* < 0.001**	0.02 (−0.00, 0.03) *P* = 0.084	0.02 (−0.00, 0.03) *P* = 0.058	−0.01 (−0.03, 0.01) *P* = 0.223	0.00 (−0.02, 0.02) *P* = 0.833
Corrected *P* value	***P* = 0.008**	*P* = 0.218	***P* = 0.001**	*P* = 0.538	***P* < 0.001**	*P* = 0.140	*P* = 0.097	*P* = 0.284	*P* = 0.880
Antenatal 26-28 weeks fasting plasma glucose (n = 653)	−0.10 (−0.27, 0.06) *P* = 0.204	0.08 (−0.07, 0.23) *P* = 0.301	0.07 (−0.09, 0.23) *P* = 0.370	0.05 (−0.11, 0.21) *P* = 0.532	0.17 (0.01, 0.32) ***P* = 0.038**	0.06 (−0.09, 0.22) *P* = 0.429	0.23 (0.07, 0.38) ***P* = 0.005**	0.17 (0.01, 0.32) ***P* = 0.037**	0.01 (−0.15, 0.18) *P* = 0.880
Corrected *P* value	*P* = 0.291	*P* = 0.502	*P* = 0.529	*P* = 0.567	*P* = 0.076	*P* = 0.429	***P* = 0.017**	*P* = 0.074	*P* = 0.880
Antenatal 26-28 weeks 2h post-OGTT plasma glucose (n = 653)	−0.05 (−0.11, 0.00) *P* = 0.057	−0.01 (−0.06, 0.04) *P* = 0.723	−0.06 (−0.11, 0.00) ***P* = 0.040**	0.02 (−0.04, 0.07) *P* = 0.502	0.00 (−0.05, 0.06) *P* = 0.905	−0.07 (−0.12, −0.02) ***P* = 0.007**	0.04 (−0.01, 0.10) *P* = 0.101	0.03 (−0.02, 0.09) *P* = 0.228	0.02 (−0.04, 0.07) *P* = 0.548
Corrected *P* value	*P* = 0.142	*P* = 0.870	*P* = 0.135	*P* = 0.567	*P* = 0.905	***P* = 0.018**	*P* = 0.127	*P* = 0.284	*P* = 0.858
Cesarean (n = 210) vs vaginal delivery (n = 443)	−0.03 (−0.19, 0.14) *P* = 0.778	0.43 (0.28, 0.59) ***P* < 0.001**	−0.04 (−0.20, 0.12) *P* = 0.616	0.20 (0.04, 0.36) ***P* = 0.017**	0.17 (0.01, 0.33) ***P* = 0.037**	−0.58 (−0.74, −0.42) ***P* < 0.001**	0.26 (0.10, 0.42) ***P* = 0.002**	0.40 (0.24, 0.46) ***P* < 0.001**	0.02 (−0.15, 0.19) *P* = 0.804
Corrected *P* value	*P* = 0.863	***P* < 0.001**	*P* = 0.684	*P* = 0.056	*P* = 0.076	***P* < 0.001**	***P* = 0.009**	***P* < 0.001**	*P* = 0.880
Female (n = 310) vs male (n = 343)	−0.09 (−0.24, 0.07) *P* = 0.265	0.10 (−0.05, 0.24) *P* = 0.181	0.13 (−0.02, 0.28) *P* = 0.083	0.17 (0.02, 0.32) ***P* = 0.029**	0.27 (0.12, 0.42) ***P* < 0.001**	0.18 (0.03, 0.33) ***P* = 0.016**	0.14 (−0.01, 0.29) *P* = 0.069	0.16 (0.01, 0.31) ***P* = 0.035**	0.13 (−0.03, 0.28) *P* = 0.115
Corrected *P* value	*P* = 0.331	*P* = 0.361	*P* = 0.208	*P* = 0.059	***P* = 0.001**	***P* = 0.031**	*P* = 0.099	*P* = 0.074	*P* = 0.288
Duration of gestation (n = 653)	0.10 (0.04, 0.15) ***P* = 0.001**	0.10 (−0.05, 0.06) *P* = 0.783	0.05 (−0.01, 0.10) *P* = 0.111	−0.09 (−0.15, −0.03) ***P* = 0.002**	0.01 (−0.05, 0.06) *P* = 0.771	−0.18 (−0.23, −0.12) ***P* < 0.001**	−0.16 (−0.22, −0.10) ***P* < 0.001**	−0.07 (−0.13, −0.02) ***P* = 0.011**	−0.09 (−0.15, −0.04) ***P* = 0.002**
Corrected *P* value	***P* = 0.008**	*P* = 0.870	*P* = 0.223	***P* = 0.013**	*P* = 0.856	***P* < 0.001**	***P* < 0.001**	***P* = 0.035**	***P* = 0.016**
Estimated fetal weight (EFW) < 10th centile < 10th centile (n = 79) vs >=10th centile (n = 574)	0.02 (−0.22, 0.26) *P* = 0.863	0.01 (−0.21, 0.24) *P* = 0.905	0.11 (−0.12, 0.34) *P* = 0.366	−0.07 (−0.30, 0.17) *P* = 0.567	0.14 (−0.09, 0.37) *P* = 0.227	0.13 (−0.09, 0.36) *P* = 0.252	−0.08 (−0.32, 0.15) *P* = 0.475	−0.06 (−0.29, 0.17) *P* = 0.620	−0.06 (−0.31, 0.18) *P* = 0.601
Corrected *P* value	*P* = 0.863	*P* = 0.905	*P* = 0.529	*P* = 0.567	*P* = 0.284	*P* = 0.315	*P* = 0.475	*P* = 0.688	*P* = 0.858

Standardized scores of cord blood TRP-KYN metabolite concentrations and ratios as outcomes. Coefficients (β) with 95% CI are change in maternal factor per standardized score change in TRP-KYN metabolite concentrations or ratios. *P* values were determined with the use of multivariable regression models. Corrected *P* values were obtained using Benjamini-Hochberg correction for multiple testing. *P* values that are significant (*P* < 0.05) are indicated in bold. Models are mutually adjusted for study site, ethnicity, maternal age, prepregnancy BMI, antenatal 26-28 weeks fasting plasma glucose, or 2h post-OGTT plasma glucose, delivery mode, intrauterine growth restriction, sex, and gestational age. Abbreviations: EFW, estimated fetal weight; HAA, hydroxyanthranilic acid; HK, hydroxykynurenine; KA, kynurenic acid; KYN, kynurenine; OGTT, oral glucose tolerance test; QA, quinolinic acid; TRP, tryptophan; XA, xanthurenic acid.

Mothers who delivered by cesarean section had higher KYN_CB_, HK_CB,_ XA_CB_, QA_CB_, and KYN/_CB_TRP_CB_ ratio, but lower HAA_CB_ concentrations, compared with mothers who delivered by vaginal delivery (Table 3). Mothers carrying female fetuses had higher umbilical cord blood concentration of HK_CB_ (115.9 [49.3] vs 107.8 [43.9] µmol/L), XA_CB_ (36.0 [14.2] vs 32.1 [13.0] nmol/L) and HAA_CB_ (785.0 [266.9] vs 742.5 [269.6] nmol/L) as well as a higher cord blood KYN_CB_/TRP_CB_ ratio compared with mothers carrying male fetuses (Supplementary Table 2b, Table 3) (22). A longer duration of gestation at delivery was associated with a higher cord blood TRP_CB_ concentration (Table 3), but lower cord blood HK_CB_, HAA_CB_, and QA_CB_ concentrations and lower KYN_CB_/TRP_CB_ and HK_CB_/KYN_CB_ ratios (Table 3). IUGR was not associated with alterations in cord blood KYN metabolites (Table 3).

### Association of Cord Blood KYN Metabolite With Neonate and Child Weight and Adiposity Measures

**Figure 1.**
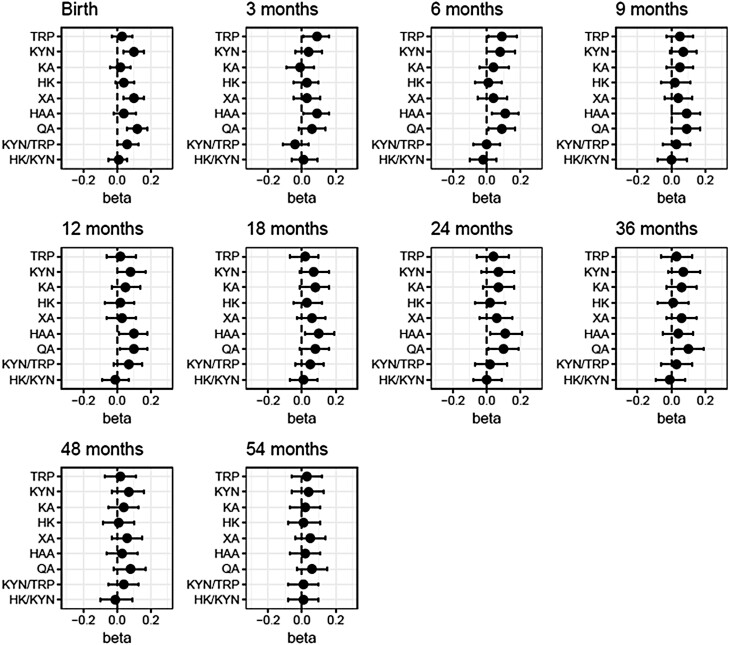
Associations of cord blood TRP-KYN metabolite concentrations and ratios with neonate and child weight. X-axes show standardized score of weights of the children over time. Forest plots show the differences (95% CI) in standardized score of weights of children from birth to 54 months with change in each standardized score of cord blood TRP-KYN metabolite or ratio (Y-axis). Models were adjusted for study site, sex, ethnicity, delivery mode, gestational age, maternal prepregnancy body mass index and antenatal fasting plasma glucose at 26-28 weeks of gestation. Total sample size (N) is not always 812 due to the missing values for covariates. Abbreviations: HAA, hydroxyanthranilic acid; HK, hydroxykynurenine; KA, kynurenic acid; KYN, kynurenine; QA, quinolinic acid; TRP, tryptophan; XA, xanthurenic acid.

In a multivariate regression model adjusting for sex, ethnicity, study site, duration of gestation, delivery mode, IUGR, prepregnancy BMI, maternal fasting glucose, higher cord blood concentrations of KYN_CB_, XA_CB_, QA_CB_ and KYN_CB_/TRP_CB_ ratio were all associated with higher birthweight (Fig. 1, Supplementary Table 4) (22). One z-score increase in KYN_CB_ and XA_CB_ was associated with a 0.10 (95% CI: 0.04, 0.16) increase in birthweight z-score, while 1 z-score increase in QA_CB_ was associated with a 0.12 (95% CI: 0.06, 0.18) increase in birthweight z-score. Although TRP_CB_ was not associated with birthweight, 1 Z-score increase in TRP_CB_ was associated with 0.09 (95% CI: 0.01, 0.18) increases in weight z-score at 3 and 6 months. Only cord blood QA_CB_ concentrations remained positively associated with weight of the child up to 3 years of age, except for at 3 months and 18 months (Fig. 1, Supplementary Table 4) (22). Although HAA_CB_ concentrations were not associated with birthweight, they were positively associated with child weight from 3 months to 2 years (Fig. 1, Supplementary Table 4 (22)).

**Figure 2.**
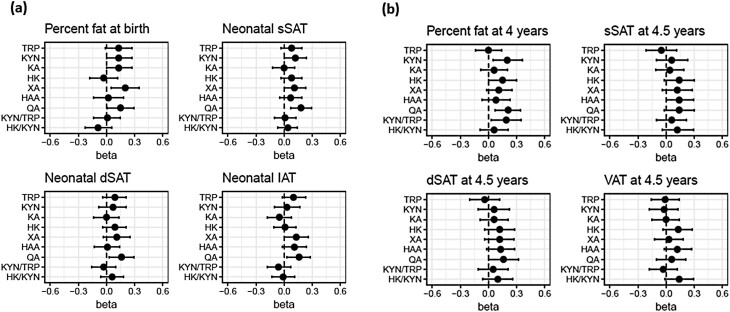
Associations of cord blood TRP-KYN metabolites and ratios with (A) neonate and (B) child adiposity. X-axes show standardized score of adiposity of the neonates and children. Forest plots show the differences (95% CI) in standardized score of fat percentage by (a) PEA POD or (b) BOD POD or abdominal adipose tissue volumes by MRI of (a) neonates and (b) children at 4 or 4.5 years with change in each standardized score of cord blood TRP-KYN metabolite or ratio (Y-axis). Models were adjusted for study site, sex, ethnicity, delivery mode, gestational age, maternal prepregnancy body mass index, and antenatal fasting plasma glucose at 26-28 weeks of gestation and day of MRI for neonatal abdominal adipose tissue volumes. Total sample size (N) is not always 255 due to the missing values for covariates. Abbreviations: dSAT, deep subcutaneous abdominal adipose tissue; HAA, hydroxyanthranilic acid; HK, hydroxykynurenine; IAT, internal abdominal adipose tissue; KA, kynurenic acid; KYN, kynurenine; QA, quinolinic acid; sSAT, superficial subcutaneous abdominal adipose tissue; TRP, tryptophan; VAT, visceral abdominal adipose tissue; XA, xanthurenic acid.

Higher cord blood XA_CB_ and QA_CB_ concentrations were associated with higher body fat percentage measured at birth (Fig. 2A, Supplementary Table 5) (22). One z-score increase in XA_CB_ was associated with a 0.20 (95% CI: 0.05, 0.35) increase in body fat percentage Z-score, while 1 Z-score increase in QA_CB_ was associated with a 0.15 (95% CI: 0.01, 0.29) increase in body fat percentage Z-score. Higher cord blood KYN_CB_, QA_CB_, and KYN_CB_/TRP_CB_ ratio were associated with higher body fat percentage measured using BOD POD at age 4 years (Fig. 2B, Supplementary Table 5) (22).

Higher cord blood QA_CB_ concentrations were associated with higher neonatal sSAT, dSAT, and IAT (Fig. 2A, Supplementary Table 5). One z-score increase in QA_CB_ was associated with a 0.18 (95% CI: 0.07, 0.29) increase in neonatal sSAT Z-score, a 0.16 (95% CI: 0.03, 0.29) increase in neonatal dSAT Z-score, and a 0.16 (95% CI: 0.03, 0.28) increase in neonatal IAT Z-score. These associations were not present at 4.5 years of age (Fig. 2B, Supplementary Table 5) (22).

## Discussion

We found that KYN_CB_ metabolite concentrations varied with neonate sex and ethnicity, maternal age, BMI, and glycemia, and with duration of gestation and mode of delivery. Concentrations of TRP and KYN metabolites were 1.6-fold (TRP_CB_) to 19.4-fold (KA_CB_) higher in the umbilical cord blood of neonates, compared to antenatal mid-pregnancy (26 week) concentrations, confirming previous observations (5, 19). Although cord blood TRP-KYN metabolite concentrations were higher than maternal concentrations, maternal and cord blood TRP-KYN metabolites were all significantly correlated with each other. This is likely due to the supply of these metabolites by active transport through the placenta (19). Higher maternal prepregnancy BMI and plasma glucose concentrations were associated with increased KYN_AN_ metabolite (KA_AN_, XA_AN_, HAA_AN_, QA_AN_) concentrations in the pregnant mother. Maternal BMI was also positively correlated with several of the analyzed KYN_CB_ metabolites (TRP_CB_, KA_CB_, XA_CB_). These findings suggest maternal health status and maternal circulating TRP-KYN are important factors in the regulation of this pathway in cord blood.

In this study, higher KYN_CB_ metabolites were found in female neonates. This is in contrast to a previous analysis in healthy young adults, where TRP-KYN metabolites tended to be lower in females (33). We further demonstrated differences between Asian ethnicities. For neonates with Indian ethnicity, we found a higher KYN_CB_/TRP_CB_ ratio relative to either Chinese or Malay ethnicities. This is suggestive of greater inflammatory activation of the catalytic steps mediated by IDO in Indian neonates. The biological significance of this sexual dimorphism and these ethnic variations are unknown; however, the GUSTO study has established the presence of heightened adiposity and early onset of increased metabolic risk in childhood for those of Indian ethnicity relative to Chinese and Malay children (30, 31, 34).

We found an inverse correlation between duration of gestation and HK_CB_, HAA_CB_, and QA_CB_. This may reflect an activation of the kynurenine pathway in the placenta in mid- to late gestation to support the antioxidant and immunosuppressive effects of HK_CB_ and HAA_CB_, and the function of QA_CB_ for NAD+ synthesis, demonstrating that the kynurenine pathway is dynamically regulated in the placenta (5, 35). However, our study only included neonates born from 30.7 to 41.4 (average 38.8) weeks; hence, we are unable to study the changes in the metabolites in early to mid-gestation. Small for gestational age (SGA) or IUGR is linked to catch-up growth and abdominal adiposity (36, 37), and associated with increased insulin resistance and cardiovascular complications in adult life (37, 38). The kynurenine pathway in the placenta has been shown to be downregulated under conditions of fetal growth restriction (39, 40). Using the common definition of IUGR based on estimated fetal weight < 10^th^ percentile (26), we did not find an association between IUGR and cord blood KYN metabolites. This may be due to the small number of neonates with IUGR (N = 79, 12.1%). Moreover, the association between cord blood KYN metabolites and birthweight and neonatal adiposity was independent of IUGR, suggesting that the positive association observed is not driven by poor fetal growth.

In examining the relationships with child weight and adiposity, several of the measured metabolites (KYN_CB_, XA_CB_ and QA_CB_) were positively associated with birthweight; however, none of the metabolites showed correlation with the weight of infants and children from 3 months to 4.5 years. Mangge et al showed that, unlike in individuals older than 18 years, the KYN/TRP ratios in overweight/obese individuals below 18 years of age were not higher than those in normal weight controls (41). Taken together, these data and the lack of association between KYN/TRP ratio and weight and adiposity in neonates and young children observed in this study suggest that IDO activity may not be a strong contributor to obesity in infancy and early childhood. A recent study showed interactions between serotonin and leptin and insulin in maintaining energy homeostasis (42). One possible link between KYN metabolites and birthweight could be via reduced maternal production of anorexigenic serotonin from TRP due to shunting to the KYN pathway.

MRI was also utilized in the first few weeks of life for the quantification of specific adipose tissue regions showing positive associations between sSAT and QA_CB_ at birth. In the GUSTO cohort, follow-up and analysis of adiposity was made again at 4 and 4.5 years using BOD POD and MRI. Interestingly, KYN_CB_, and QA_CB_ were correlated positively with total body fat percentage measured by BOD POD at age 4. There is little evidence of QA possessing a function that may explain its positive relationship of elevated cord blood levels and heightened adiposity in the growing child. While QA is potentially neurotoxic by acting as a NMDA receptor agonist, this action is possibly counterregulated by KA that exhibits a neuroprotective NMDA receptor antagonist (1, 17, 18). Possibly of greater importance to whole-body energy homeostasis is that QA is also the precursor for the synthesis of NAD^+^ (1, 3). It would be of interest to relate cord blood QA to measures of appetite in the children. Exogenous factors such as diet likely play a major role for the lack of influence of cord blood KYN metabolites on later child weight and adiposity in early life. These data suggest a possibility of KYN_CB_ and QA_CB_ as biomarkers of early childhood adiposity, although further studies are required to confirm this hypothesis.

There are several important limitations and considerations in the interpretations of this data. First, analysis was performed on whole cord blood; therefore, there is no capacity to distinguish the differences in TRP-KYN metabolites in venous and arterial supply. Further, our analysis did not include measurement of maternal KYN metabolite concentrations at the time of delivery. TRP and KYN metabolite concentrations have been demonstrated to change over pregnancy (43). While total TRP was shown to decrease during pregnancy, free TRP was shown to increase in pregnancy (44). Of note in our data are the significant correlations between the metabolites from samples collected from the mothers at mid-pregnancy (week 26-28) and neonatal cord blood metabolites. Further, no measurement was made of the subsequent concentrations of TRP-KYN metabolites at the same time as the measurements of body composition in the growing infant. Therefore, we and others have a limited understanding of how cord concentrations are predictive of early life TRP-KYN metabolite levels and how these change from the first to fourth years of life. These longitudinal analyses are required. In addition, information on diet and medication use records specifically for tryptophan were not available. As dietary factors and medications may potentially contribute significantly to circulating metabolites, it is possible that the observed associations may be confounded by diet or medications. Moreover, differences between those with fat percentage/abdominal adipose volumes measured and those without these measurements can introduce bias to the findings and interpretation. Furthermore, due to small sample sizes, particularly for neonates and children with fat percentages and abdominal adipose tissue volumes measured, insignificant results could be due to insufficient power. Lastly, critical to this study is the requirement for additional insights into the specific biological functions of each KYN metabolite, particularly in relation to adiposity and metabolic health risks.

## Conclusion

KYN_CB_, XA_CB_, and QA_CB_ were positively associated with birthweight, while QA_CB_ was positively associated with neonatal superficial abdominal adipose tissue volume. KYN_CB_ metabolites (XA_CB_ and HAA_CB_) were higher in female infants and demonstrated association with ethnicity. Cord blood KYN metabolites concentrations were associated with maternal concentrations, and both higher maternal BMI and higher antenatal glucose concentrations were associated with higher cord blood levels. Of the measured KYN_CB_ metabolites, KYN_CB_ and QA_CB_ positively associated with body fat percentage at 4 years, suggesting a possibility of these cord blood metabolites as early biomarkers of later whole-body adiposity.

## Data Availability

Datasets generated during and/or analyzed during the current study are not publicly available but are available from the corresponding author on reasonable request.

## Abbreviations

AA: anthranilic acid
BMI: body mass index
dSAT: deep subcutaneous adipose tissue
EFW: estimated fetal weight
GUSTO: Growing Up in Singapore Towards healthy Outcomes
HAA: 3-hydroxyanthranilic acid
HK: 3-hydroxykynurenine
IAT: internal adipose tissue
IDO: indoleamine-2,3-dioxygenase
IUGR: intrauterine growth restriction
KA: kynurenic acid
KMO: kynurenine-3-monooxygenase
KYN: kynurenine
KYN_AN_: antenatal KYN
KYN_CB_: cord blood KYN
LC-MS/MS: liquid chromatography–tandem mass spectrometry
MRI: magnetic resonance imaging
NAD^+^: nicotinamide adenine dinucleotide
NMDA: N-methyl-D-aspartate
OGTT: oral glucose tolerance test
QA: quinolinic acid
sSAT: superficial subcutaneous adipose tissue
TDO: tryptophan-2,3-dioxygenase
TRP: tryptophan
VAT: visceral adipose tissue
XA: xanthurenic acid
